# Eccentric muscle contractions: from single muscle fibre to whole muscle mechanics

**DOI:** 10.1007/s00424-023-02794-z

**Published:** 2023-02-15

**Authors:** André Tomalka

**Affiliations:** Motion and Exercise Science, University of Stuttgart, Stuttgart, Germany

**Keywords:** Contractile behaviour, Stretch, Muscle physiology, Skeletal muscle, Titin, Sarcomere

## Abstract

Eccentric muscle loading encompasses several unique features compared to other types of contractions. These features include increased force, work, and performance at decreased oxygen consumption, reduced metabolic cost, improved energy efficiency, as well as decreased muscle activity. This review summarises explanatory approaches to long-standing questions in terms of muscular contraction dynamics and molecular and cellular mechanisms underlying eccentric muscle loading. Moreover, this article intends to underscore the functional link between sarcomeric components, emphasising the fundamental role of titin in skeletal muscle. The giant filament titin reveals versatile functions ranging from sarcomere organisation and maintenance, providing passive tension and elasticity, and operates as a mechanosensory and signalling platform. Structurally, titin consists of a viscoelastic spring segment that allows activation-dependent coupling to actin. This titin-actin interaction can explain linear force increases in active lengthening experiments in biological systems. A three-filament model of skeletal muscle force production (mediated by titin) is supposed to overcome significant deviations between experimental observations and predictions by the classic sliding-filament and cross-bridge theories. Taken together, this review intends to contribute to a more detailed understanding of overall muscle behaviour and force generation—from a microscopic sarcomere level to a macroscopic multi-joint muscle level—impacting muscle modelling, the understanding of muscle function, and disease.

## Introduction

### Versatile muscle functions in eccentric loading

**Fig. 1:**
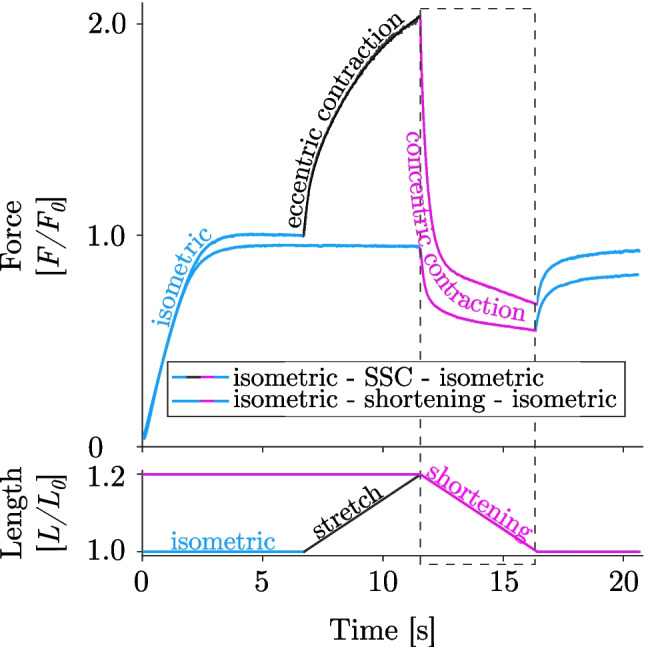
Representative force-time (upper row) and length-time (lower row) trace of a single skinned EDL muscle fibre (*n* = 1) performing a stretch-shortening-cycle (SSC, black-magenta line) and a pure shortening contraction (magenta line). The mechanical work is significantly larger for the SSC condition compared with the active shortening condition (cf. dashed rectangle)

Skeletal muscles represent fascinating and complex machinery, enabling active force production, movement and stability of the skeleton, storage and transport of substances within the body, and generation of heat (1, 2). These multiple functions are based on the way muscles work. Muscles perform concentric, isometric, and eccentric contractions and the combinations thereof. The fundamental understanding of skeletal muscle contraction is central to muscle physiology. While the molecular and cellular mechanisms underlying concentric (force generation during muscle shortening) and isometric (at constant muscle length) contractions are quite well described by the classic sliding-filament (3, 4) and cross-bridge theories (5), at least for a particular length range (6), the mechanisms underpinning eccentric contractions remain to be elucidated. Eccentric contractions refer to muscle actions that occur when the external force applied to the muscle exceeds the force produced by the muscle itself, resulting in a lengthening action (i.e. when work is done on the muscle) (7, 8). Lengthening actions are an essential part of everyday movements involving deceleration, e.g. after a jump or walking downstairs, support the weight of the body against gravity, serving as shock absorbers and struts during locomotion (9). Another important feature of eccentric contractions is their ability to absorb mechanical energy during muscle lengthening, recover that stored energy and increase the active force generated during subsequent shortening contractions compared to pure muscle shortening (10, 11). This coupling of eccentric immediately followed by concentric contractions is referred to as stretch-shortening cycles (SSCs; Fig. 1)—an important phenomenon associated with powerful and efficient movements at reduced metabolic energy expenditure (11–13).

Compared to concentric and isometric muscle actions, eccentric contractions show some further unique features responsible for the high efficiency observed. Fewer motor unit activation (14, 15), reduced cardiorespiratory and hemodynamic reactions, and less metabolic consumption for a given force (7), are associated with eccentric muscle actions (9, 16). These features yielded a growing interest over the last decades. Particularly in light of the health-related effects, an increased number of investigations focused on the beneficial outcomes of eccentric resistance training (for reviews see (9, 17, 18)). But how exactly can muscles exert high forces with little energy expenditure during lengthening actions? Two major sources have been proposed to account for unexplained observations during and after eccentric contractions, *(I)* the nervous system (14, 15) and *(II)* the muscle itself (19, 20). While recently published evidence has greatly improved our understanding of the control strategies driven by the nervous system during muscle lengthening (i.e. reduced spinal and corticospinal excitability; for reviews see (14, 15)), a substantial gap remains in our understanding of the cellular and molecular mechanisms underlying eccentric loading, in particular during long stretch contractions.

There are becoming piles of literature on the mechanosensing contributions of cross-bridge activation and force-dependent cross-bridge recruitment upon muscle stretch in the past decade (21–25). Moreover, several model approaches (26–29) and ample experimental evidence (30, 31) point to a strong contribution of the giant protein titin to force generation during and after eccentric contractions.

Therefore, this review aims to discuss recent investigations—from single muscle fibre to whole muscles—to explain the mechanistic basis underlying eccentric contractions. Additionally, this article intends to underscore the functional link between sarcomeric components, emphasising the role of titin in skeletal muscle.

## Physiological and mechanical phenomena associated with eccentric muscle loading

I. *Force-velocity-relation*

**Fig. 2:**
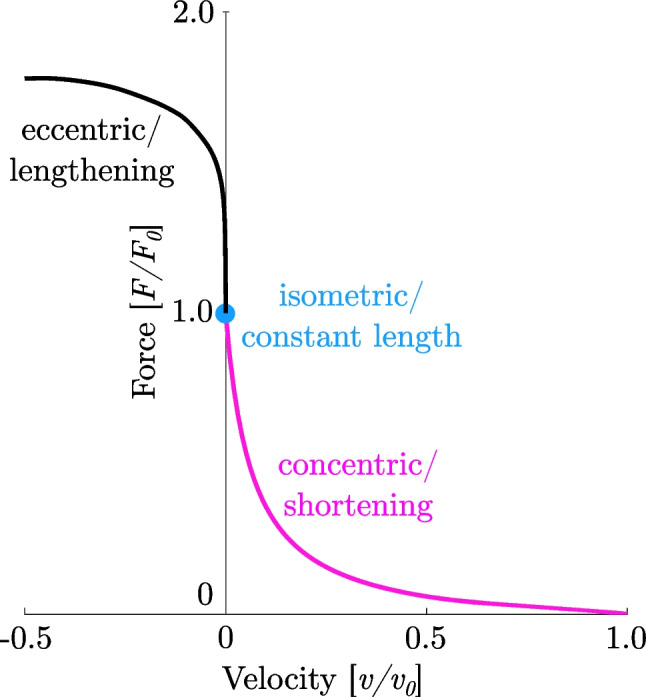
Representative sarcomere force-velocity relationship (*FVR*)—illustrated by a maximally Ca^2+^-activated skinned single fibre of a rat soleus muscle (*n* = 1). The experiments are conducted at a constant temperature of 12°C. The magenta curve shows the typical hyperbolic shape of the concentric *FVR* observed by Hill (32). The black curve depicts the eccentric *FVR* during active muscle lengthening. Velocity is zero at maximum isometric force *F*_*0*_ (blue dot)

**Fig. 3:**
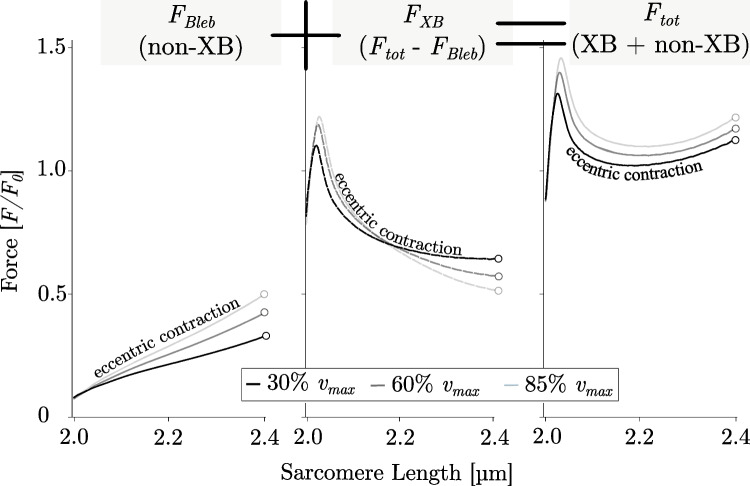
Different contributions of XB- and non-XB-components to total muscle force during eccentric contractions. Eccentric force-sarcomere length traces were obtained in SSC contractions with varying velocities. Data reproduced from (12). Lines depict mean force values obtained by skinned single muscle fibres of rat soleus muscles at 30% *v*_*max*_ (black), 60% *v*_*max*_ (medium grey), and 85% *v*_*max*_ (light grey) (*n* = 13 fibres from five rats). *F*_*Bleb*_; depressed force induced by the XB-inhibitor Blebbistatin, *F*_*XB*_; ‘Isolated XB’ forces depict the difference between the total force *F*_*tot*_ and *F*_*Bleb*_ (11). Consequently, the total force *F*_*tot*_ is composed of non-XB structures (mainly titin) and XB-structures (contractile proteins like actin and myosin)

**Fig. 4:**
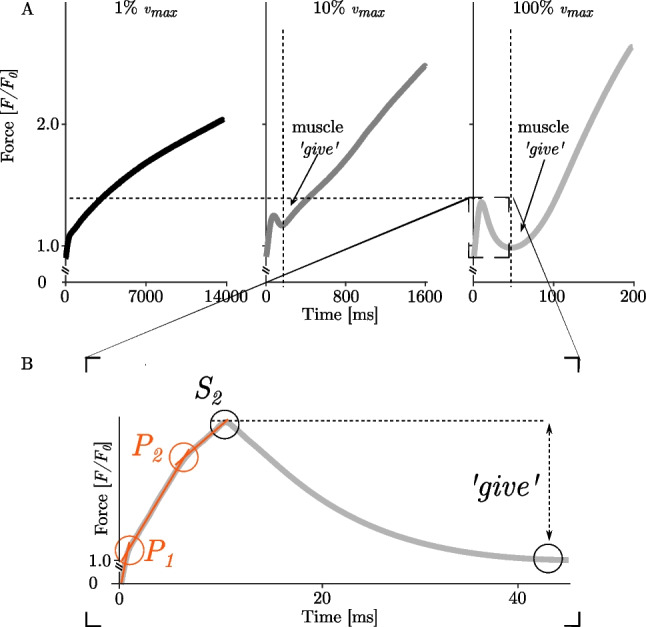
**A** Force-time plots of eccentric ramp experiments of skinned single muscle fibres of rat EDL muscles at 1% *v*_*max*_, 10% *v*_*max*_ and 100% *v*_*max*_ (from left to right). Data reproduced from (40). The fibres were stretched from about 2.0 to 2.9 µm sarcomere length. The black (*n* = 18), mid grey (*n* = 7) and light grey lines (*n* = 8) depict mean values. The vertical dashed lines represent the point in time where force reaches a local minimum. The horizontal dashed line indicates the increase in *S*_*2*_ with increasing stretch velocity. **B** Zoom in on the initial force response upon eccentric contractions. Muscle ‘*give*’ is defined as the difference between the first local force maximum (*S*_*2*_) and the force minimum. *P*_*1*_ and *P*_*2*_ depict characteristic transitions during the initial rise in force following stretching

A fundamental principle of skeletal muscle physiology and a main determinant of muscle force production is the force-velocity relationship (*FVR*). The concentric (shortening contractions) part of the *FVR* has been first observed and described mathematically based on pioneering studies on isolated frog muscles by Hill (32). The *FVR* describes the relation between the maximum muscle force and its instantaneous rate of change in length. If a muscle shortens during contraction, the shortening velocity depends on the load, while the contraction velocity decreases with increasing load in a hyperbolic manner (23; Fig. 2 magenta line). On the contrary, the *FVR* upon muscle stretch does not follow the classic hyperbolic *FVR* for concentric contractions (Fig. 2 black line) (32, 33). The ability to exert high maximum forces during lengthening contractions is much less at slow velocities compared to fast eccentric velocities. However, there is a much greater potentiation of force (ranging from 1.0 to 1.5 *F*_*0*_) at very slow lengthening velocities with only marginal change in velocity (less than 0.1 *v*_*0*_). In this range, the eccentric part of the *FVR* was observed to be nearly constant (Fig. 2, almost vertical section of black line). In contrast, at forces between 1.5 and 1.8 *F*_*0*_, velocity changes were progressively larger, with smaller increases (and plateauing) in force (34, 35). Early studies by Katz (36) on electrically stimulated sartorius muscles from frogs found that the force produced by the muscle during rapid lengthening exceeded the isometric force (Fig. 2 blue dot) substantially by factor ×1.8 *F*_*0*_. Thereby, the slope in force is significantly greater (by factor ×4–6) for eccentric than for concentric contractions (36, 37). This observation is consistent with recent studies on intact and skinned muscle samples (mammalian and amphibian) over a wide range of velocities (12, 38–40). The experimental findings of these studies demonstrated an increase in peak force in eccentric muscle loading as a function of increasing stretching velocity (Fig. 3 right plot, Fig. 4A).

This muscle behaviour strongly deviates from the classic hyperbolic shape of the *FVR* observed for concentric contractions and depicts a distinct feature of eccentric contractions (cf. magenta and black line of Fig. 2). Classic Hill-type model approaches (32, 33) suggest cross-bridges (XBs) (formed by actomyosin interaction) as the only activation-dependent force-generating components in muscles. However, recent studies show that there is an additional parallel ‘non-cross-bridge’ (non-XB) component (e.g. titin) contributing to the total force response—particularly during eccentric muscle loading (12, 26, 31, 39). Consequently, both XB and non-XB components are involved in eccentric contractions (Fig. 3).

Anyhow, the proportion of their contributions to force production, especially the role of titin in the *FVR*, is not yet clear (12). Contrary to the prevailing assumption that XB-forces increase with increasing stretch velocities up to a certain level (33, 41), recent results by Tomalka et al. (12) showed decreasing XB-forces for increasing stretch velocities at the end of the lengthening contraction (Fig. 3 middle plot). They performed in vitro isovelocity ramp experiments with varying ramp velocities (30, 60, and 85% of maximum contraction velocity [*v*_*max*_]) on single-skinned soleus muscle fibres from rats. The different contributions of XB (Fig. 3 middle plot) and non-XB structures (Fig. 3 left plot) to total force production (Fig. 3 right plot) were identified using the XB-inhibitor Blebbistatin. This photosensitive chemical has rather complex actions on XB function. As suggested by (42, 43), Blebbistatin inhibits the force-producing transition of the bound actomyosin complex that traps myosin heads in a weakly actin-attached state without exerting any force (44). However, long stretches applied to muscles treated with Blebbistatin are likely to result in detachment of weakly bound XBs, inferring only a marginal XB contribution to the force response upon muscle stretch. These findings suggest a central role for titin in the eccentric *FVR* (for a detailed review see (35))*.*

II. *Muscle ‘give’*

Numerous studies have shown that fibre kinetics in eccentric muscle loading are characterised by a steep rise in force during the early phase of the stretch, immediately followed by a relatively compliant transient phase (Fig. 4A, middle and right subplot). The initial linear phase (Fig. 4B, orange lines) is biphasic with a steep force slope (*P*_*1*_, Fig. 4B) followed by a more gradual change in slope (*P*_*2*_, Fig. 4B) (39). This observation is following recent investigations of stretch-induced force responses (5% *L*_*0*_ stretch amplitude) in intact and skinned muscle fibres across a wide range of velocities (38–40, 45–47). Both transitions have been related to XB characteristics and are attributed to the extension of all attached myosin heads to actin (12, 37, 39, 40, 45). *P*_*1*_ (termed *S*_*1*_ by Flitney & Hirst (48)) occurs in a short time immediately after the onset of the stretch between 0.14–0.20% *L*_*0*_ (39). *P*_*2*_ is reached for extensions of 1.16–1.34% *L*_*0*_ (39, 48). Recent experimental findings suggest that the transition *P*_*1*_ is mainly due to the extension of all originally attached myosin heads, while *P*_2_ occurs when the ‘switchover from original to new heads is essentially complete’ (39). Thus, between the *P*_*1*_ and *P*_*2*_ transitions, the original myosin heads quickly detach, leaving only the newly attached heads. Recent findings demonstrate that the shape of the eccentric force response changes with increasing stretch velocity (40, 49) (Fig. 4A). The impact of stretch velocity has been observed in a series of studies for comparatively short stretch amplitudes (mainly about 2% to 5% *L*_*0*_). The force occurring at both transitions increased with increasing stretch velocity (37, 39, 40). Continuous stretching beyond *P*_*2*_ resulted in a force peak (*S*_*2*_, Fig. 4B; (48)) followed by a negative force slope until the force recovers by the end of the stretching phase. *S*_*2*_ also increased with increasing stretch velocity (12, 39, 40, 48). The phase of negative force slope after *S*_*2*_ was termed muscle ‘*give*’ (Fig. 4) (48). This term refers to the displacement of the filaments in the axial direction exceeding 11–12 nm, while the XBs are forcibly detached (50–52) and sarcomeres are no longer able to resist the rise in force upon active muscle lengthening (48).

These findings are supported by a previous study by Tomalka et al. (12). They investigated the effect of varying velocity of the length changes during SSCs on the power output in skinned fibres of rat soleus muscles. Additionally, they used the molecular myosin inhibitor Blebbistatin to differentiate between XB and non-XB contributions to the mechanical responses. They found increased power output with increased SSC-ramp velocities. Based on evidence for increased storage and release of energy in non-XB conditions, the authors conclude that energy stored in titin during eccentric contraction contributes to the increase in power output with increased velocity. For all tested velocities in the control experiments (30%, 60%, 85% *v*_*max*_), fibre kinetics were characterised by muscle ‘*give*’ during the stretching phase of SSCs ((12), their Fig. 4). In the presence of Blebbistatin (20 µmol l−1), it has been found a quasi-linear force response during the SSCs’ stretch phase for all tested velocities ((12), their Fig. 5) with no muscle ‘*give*’ upon active stretching. However, it cannot be taken for granted that Blebbistatin completely eliminates XB-based force production, since Blebbistatin (and similar drugs as butanedione monoxime (BDM) (53, 54) and benzyl-toluene sulfonamide (BTS) (37)) seems to affect the contractile apparatus in a complex manner (42, 55, 56). There are indications that Blebbistatin leads, among other things, to a considerable reduction of *v*_*max*_ under certain conditions (42, 57). An effect that is explainable by the potential influence of an increased population of weakly bound XBs, which are suggested to contribute to an increase in stiffness and non-XB-based force while strained during muscle stretch (39, 42, 53, 56). Consequently, regardless of the effect of Blebbistatin on the contractile apparatus, a contribution of weakly bound XBs to force during the stretch (53, 56) seems to be likely for small stretch amplitudes (≈ 1.5% *L*_*0*_) only. For rather extensive ramp amplitudes weakly bound XBs rapidly detach (58, 59), so the strain of XBs only contributes to the initial rise in force.

**Fig. 5:**
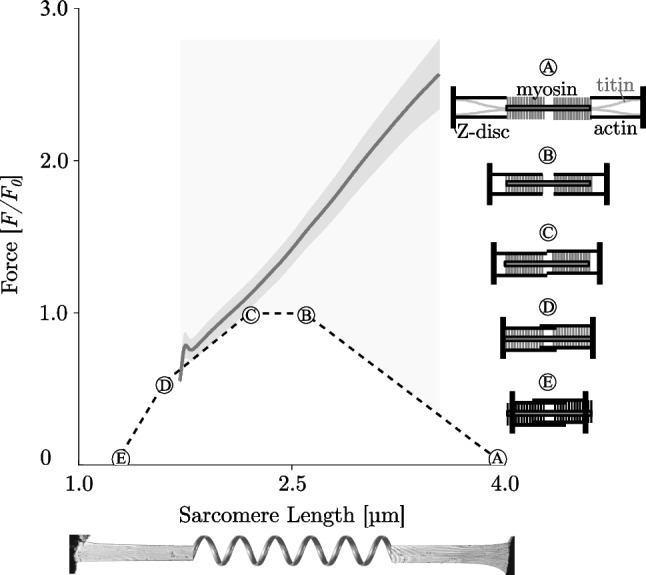
Extensive ramp contractions with a stretch amplitude of 0.75 *L*_*0*_ show a linear increase in force. The solid line depicts the mean and the shaded regions around the solid line indicate the corresponding s.d. during active stretching. Measurements of skinned skeletal fibres from EDL muscles are shown. Data reproduced from (30). The active isometric *FLR* can be directly explained by actin and myosin filament overlap. Quantitative changes in overlap (see corresponding sarcomere configuration schematics (A)–(E) to the right) lead to slope changes of the *FLR*. Bottom: representative picture of a permeabilized single muscle fibre of a rat EDL working like a linear spring

Muscle ‘*give*’ occurred during stretch amplitudes of 1.2 to 2.1% optimal sarcomere length (32, 34–37). Therefore, ‘*give’* would be expected when XBs contribute to force production during sufficiently long eccentric ramps. Continuous muscle stretching beyond the local force minimum results in a force redevelopment (Fig. 4A, force-time traces to the right of the vertical lines). This rise in force is attributed to the continuous stretch of non-XB elements (39, 60). Thus, elastic energy stored in viscoelastic structures, such as titin, increases with increasing stretching velocity (39, 61, 62)—suggesting an important role for titin, particularly in long eccentric contractions (12, 37, 39, 40, 63). Consequently, XB dynamics seem to dominate the first part of the stretch, while titin dynamics dominate the second part.

III. *Force enhancement—muscles act like linear springs*

A quasi-linear increase in force during eccentric ramp contractions (force enhancement, FE) for small to moderate changes in length (2–20% *L*_*0*_) has been reported by several studies (12, 37, 39, 64–66). More recently, experimental observations on rats demonstrated a spring-like behaviour of single muscle fibres from the musculus extensor digitorum longus (EDL) during long, eccentric contractions of 0.45 *L*_*O*_ (30, 40) and 0.75 *L*_*0*_ (30)—nearly over the entire force-length-relation (*FLR*)*.* Thereby, muscle forces up to ×2.5 of the maximum isometric force can be generated (Fig. 5). This exceeds the maximum active forces produced by XBs at these lengths, which is in strong contrast to the classic isometric *FLR*. This is a surprising result because the underlying non-linear *FLR* consists of linear segments that decrease in slope with increasing fibre length. These changes in slope are the result of variations in myofilament overlap (Fig. 5, sarcomere configuration schematics (A)–(E)). The classic sliding filament (3, 4) and XB-theories (5) cannot explain these observations. Ignoring the low passive force up to 3 µm, the classic theories predict that, aside from an initial force increase, the force during an isovelocity stretch follows the shape of the *FLR* scaled with a factor greater than one (due to the eccentric *FVR*). In particular, this means that the expected force in the plateau should be constant, and should decline on the descending limb due to a decreasing number of available XBs. In contrast, in the second half of the stretch (Fig. 5) the force is constantly increasing. In addition, the positive slopes increase with velocity instead of decreasing as the classic theories would predict. Thus, for long-stretch contractions, the active isometric *FLR* is no longer visible during eccentric muscle loading. Moreover, it could be demonstrated that both XBs and non-XBs contribute nonlinearly to the resulting linear total muscle force response (30). The spring-like and viscous non-XB effects observed are likely attributed to titin. This suggestion is in line with recent work (67, 68), which measured heat production and force of muscle fibres from frogs during ramp stretches. They suggested that XBs account for only ≈ 12% of the total energy storage during the active stretch. Accordingly, more than 85% of energy storage upon muscle stretch cannot be explained by XB mechanisms, particularly since attached XBs detach quickly from actin filaments (50), and their stored elastic energy is lost (12, 69, 70).

IV. *Residual force enhancement*

**Fig. 6:**
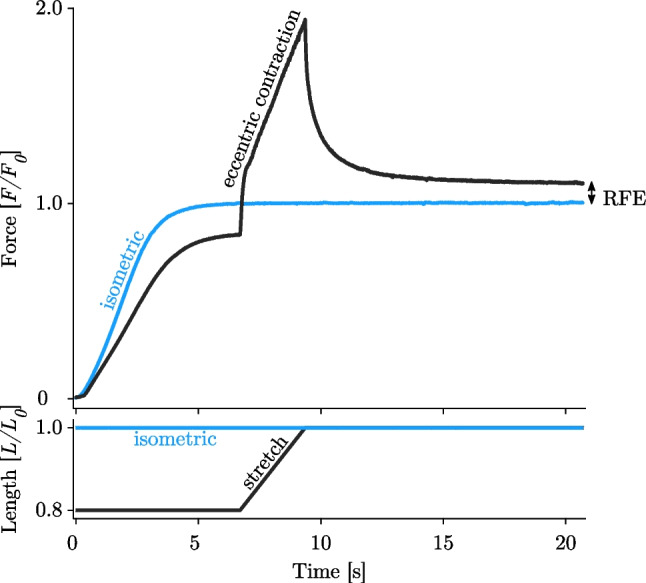
Representative force–time (upper graph) and length–time traces (lower graph) of a skinned single EDL muscle fibre (*n* = 1). The fibre is maximally Ca^2+^-activated (*pCa* = 4.5) at *t* = 0 s for 21 s. The blue line is the isometric reference contraction at optimum fibre length 1.0 *L*_*0*_. The black line depicts an eccentric contraction from 0.8 to 1.0 *L*_*0*_ (between 7 and 9.5 s). The force is enhanced by about 10% *F*_*0*_ in the isometric steady-state phase after the active stretch compared to the pure isometric force (RFE). The stretch velocity is 0.1 *L*_*0*_*/s*

**Fig. 7:**
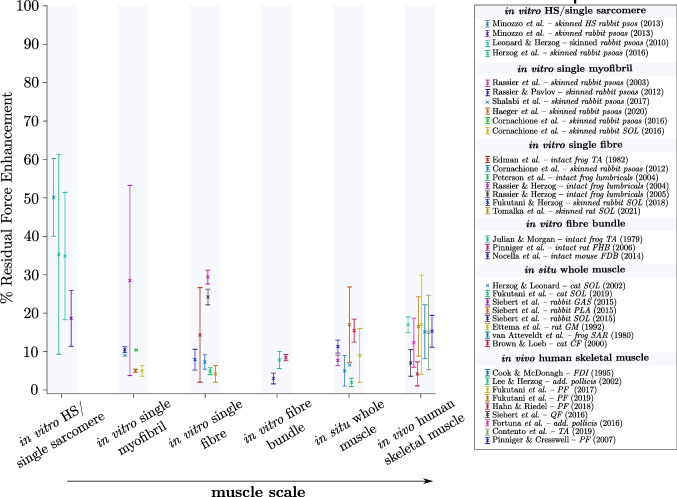
Overview of the magnitude of residual force enhancement in percent [%] of various mammalian and amphibian muscles from the literature, categorised by the muscle structural level. Note that only statistically significant values different for pure isometric reference contractions and steady-state isometric force/torque after stretch are included. To enable comparisons between experiments on animal and human muscles, all data are for electrically evoked contractions or calcium-activated samples (skinned preparations). For studies reporting different stretching velocities, activation levels, or stretch amplitudes, data were pooled to generate a mean with a corresponding standard deviation (error bars). SOL, musculus soleus; GAS, musculus gastrocnemius; PLA, musculus plantaris; GM, musculus gastrocnemius medialis; SAR, musculus sartorius; CF, musculus caudofemoralis; FDI, first dorsal interosseus; PF, plantar flexors; QF, musculus quadriceps femoris; TA, musculus tibialis anterior

A further distinguishing feature of eccentric muscle loading is that striated skeletal muscles generate higher active forces after stretch (residual force enhancement, RFE) if compared to the muscles’ corresponding isometric force at constant length (Fig. 6). This fact has been known for about 70 years (71) and has been investigated across all muscle structural levels, from in vitro isolated (half-) sarcomeres (72–74), myofibrils (75–79), single muscle fibres (47, 54, 80–84), muscle fibre bundles (39, 85, 86), single muscles (49, 66, 87–90) and in vivo single and multi-joint movements ((91–99); for review see (100)). RFE increases with the amplitude of stretch (61, 71, 80), and is almost independent of stretch velocity (101, 102), except for fast stretch velocities associated with muscle ‘*give*’ (forcibly detachment of attached XBs, see chapter *II. Muscle* ‘*give*’) (12, 40, 48, 49). (R)FE is long-lasting (minutes in skinned fibres and single myofibrils) and can be stopped immediately by deactivating the muscle (71, 103). However, RFE observed in the activated muscle often persisted following deactivation in the passive muscle, which is called passive force enhancement ((87, 92, 104–106); for a detailed review see (107)). Additionally, RFE exists at almost all muscle lengths (82, 108). Although experimental data in the literature are somewhat controversial regarding the appearance of RFE in different regions of the *FLR*. While some studies show that RFE exists at all muscle lengths (82, 87), other studies show little or no RFE on the ascending limb of the *FLR* (61, 104). However, there seems to be general agreement in the literature that the magnitude of RFE is greatest, particularly in the range of the descending limb of the *FLR* (26, 109, 110). Figure 7 shows an overview of the magnitude of RFE scaling with muscle size. Despite a high inter- and intraindividual variability of the data compared, there is an apparent trend for the decrease of RFE in magnitude—at least for in vitro muscle samples—from the smallest functional contractile unit of the muscle (the (half-) sarcomere) towards isolated muscle fibre bundles. Despite clear evidence of RFE across all structural muscle levels, the contraction modalities and applied methods (such as e.g. the stretch amplitude, contraction velocity, activation levels and experimental temperature, studied animal model, and titin isoform) might have important implications for experimental findings of comparable studies in the literature. These different methodological boundary conditions likely explain considerable variability in stretch-induced force responses ((R)FE). Since the structural and mechanistic complexity increases with muscle size, it is challenging to compare the findings of in vitro animal studies with multi-joint muscle actions in vivo. Therefore, when analysing RFE at different muscle levels, the superposition of multiple effects (e.g. interaction with surrounding tissues, synergistic muscle actions, three-dimensional muscle architecture [pennation angle, fascicle rotation], complex activation patterns, a mixture of muscle fibre types, neuromuscular fatigue) should be considered (111, 112).

Although a substantial gap remains in our understanding of mechanisms underlying eccentric contractions (113, 114), (R)FE is a well-acknowledged and fundamental property of muscle behaviour (26, 74, 113, 115, 116).

V. *Ultrastructural changes*

Besides the well-known benefits, eccentric muscle contractions have been associated with potentially inducing various damage on the muscle fibre contractile and cytoskeletal structures. High mechanical loading of involved tissue structures can lead to microlesions and partial necrosis, disruption of the excitation-contraction coupling or the extracellular matrix, Z-disc streaming, sarcolemma damage, swelling of mitochondria, dilatation of the T-tubule system, and delayed onset muscle soreness [DOMS]. These degenerative changes have several functional consequences for health and disease and were suggested to contribute to muscle weakness in Duchenne muscular dystrophy (117), or after eccentric exercise (118). These topics have been thoroughly reviewed recently (8, 9, 119) and will not be further discussed here.

## Cellular and molecular mechanisms

i. *The role of mechanosensitive properties of cross-bridges*

A potential mechanism to explain eccentric muscle behaviour within the sarcomere might be explained with force-dependent or stress-dependent XB recruitment upon stretch. The thick filament operates as a regulatory mechanosensory for the regulation of force generation in skeletal muscles (22, 120, 121). Recent X-ray diffraction studies on actively contracting fibres from striated skeletal muscle (21, 22, 25, 122) suggest that the myosin filament can exist in one of two possible states: a relaxed state (OFF) and an activated state (ON). In the ‘OFF’ (22) or ‘super relaxed’ state (123), seen in resting muscle, the great majority of myosin motors is made unavailable for actin binding or ATP hydrolysis (22). However, a small fraction of ON motors ‘allows the muscle to respond immediately to calcium activation when the external load is low (22)’. At high loads, the myosin filaments are switched ON by mechanical stress due to stretch-dependent activation accompanied by the mobilization of more myosin motors that generate more force (21, 22). These results suggested that this regulatory mechanism of thick filament mechanosensing in striated muscles acts independently of the well-known thin filament-mediated calcium-signalling pathway (21) and might have broad implications on the force generation in lengthening contractions (21). However, for long magnitudes of stretch, the majority of XBs are likely to detach (see chapters *II and III*), while only a fraction of bound XBs is capable to contribute to enhanced forces observed during long eccentric contractions (12, 30, 40, 67, 68). There are several hints that these enhanced forces are due to increased non-XB forces. A series of experiments, in which XB formation is hampered by actomyosin inhibitors, enabled the estimation of non-XB contributions to FE (30, 78, 124, 125).

To date, there is no single accepted mechanism that explains the high force generation upon muscle stretch. A recent study by Fusi et al. (21) suggests a possible role of titin in the regulation of muscle contractility due to thick filament activation mediated by the mechanosensory pathway in the myosin filament (22, 126).

ii. *The role of titin*

Albeit extensive experimental research has been done on isolated muscles for over 100 years (127), underlying force-generating mechanisms are not fully understood at this time. Even the generally accepted and groundbreaking Hill (32) and Huxley-type models (3–5) are not capable to describe muscle force during and after eccentric contractions.

Despite several explanatory approaches for the unique properties of eccentric muscle loading, no generally accepted model exists. Mechanisms discussed include modified XB kinetics (128, 129), the contribution of sarcomere length dynamics (80, 115, 116, 130, 131), and non-XB contributions to muscle force (103). Some of these mechanisms or explanations can be partially ruled out based on the following criteria. The proposed modifications of the XB cycle have not yet been confirmed experimentally (132). Moreover, only a fraction (0.05 *F*_*0*_) of the experimentally observed dynamics can be described by sarcomere length inhomogeneities (104, 115). Other authors prefer explanatory approaches in which non-XB structures play a crucial role in eccentric muscle loading (113, 133). In addition to the XB components (containing contractile [actin and myosin] and regulatory proteins [i.a. troponin, tropomyosin]), muscle fibres consist of several non-XB components (containing structural proteins [i.a. titin, nebulin, desmin]). These structural proteins have versatile and complex functions in muscle contraction. They contribute to stability, elasticity, alignment, and even active force production—although in a supportive manner (133). Titin is known to play a key role in eccentric muscle loading and has an integral function as a modulator of muscle contraction (133). Functionally, this protein has been reported to serve as a scaffold for the biogenesis of sarcomeres (134), alignment of the myosin filament (133), maintenance of sarcomere length (135), and preservation of passive force and (visco-)elastic recoil in the sarcomere (12, 134). Titin also modulates the actin-myosin-based force production via non-XB formation (26, 30, 136).

**Fig. 8:**
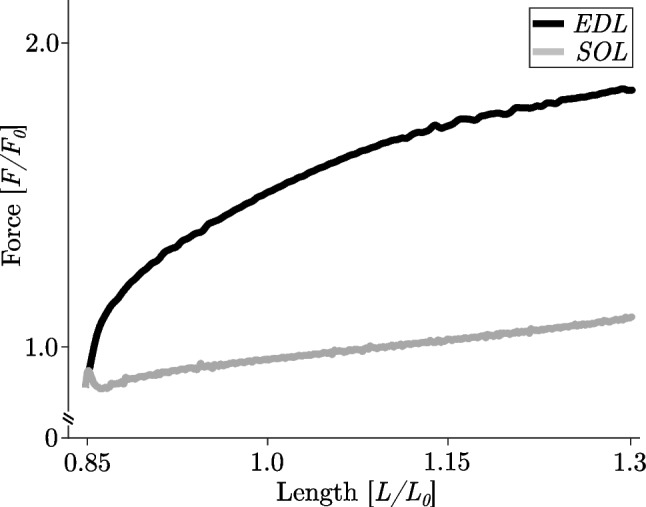
Representative trajectories of force development in a slow muscle fibre (soleus, SOL; grey line) and fast muscle fibre (extensor digitorum longus, EDL; black line) during eccentric contractions with comparable stretch velocity (1.5% *v*_*max*_) and amplitude (0.45 *L*_*0*_). After an initial increase in force (short-range stiffness (180)), the SOL fibre shows a pronounced yielding (muscle ‘*give*’ (48)). In both muscle fibres, the force increases monotonically during slow stretch contractions, while the force response of the EDL fibre is about an order of magnitude larger at the end of the stretch

Structurally, skeletal muscle titin is referred to as the third myofilament and is the most abundant protein in skeletal muscles with a molecular mass between 3800 and 4200 kDa (137, 138). Titin spans half a sarcomere from the Z-disc to the M-line. It firmly anchors to myosin in the A-band region and then runs freely across the I-band region of the sarcomere until it attaches to actin (approx. 50–100 nm away from the Z-disc) before finally entering the Z-disc. Thereby, this giant protein forms a ‘permanent’ interconnection between the thin and thick filaments of muscle sarcomeres (133, 136, 139). This filamentous protein consists of two segments, a free spring segment located in the sarcomeric I-band with highly variable elastic properties (133, 140) and a less compliant part of titin in the A-band (141). Titin at the Z-disc, A-band, and M-band has primarily structural roles by binding to other main components of the sarcomere (i.a. α-actinin and actin at the Z-disc, myosin heavy chain protein and myosin-binding protein C in the A-band, and myomesin within the M-band) (133, 142). It effectively interacts with more than 30 muscle proteins. Titin’s I-band consists of a proximal and distal immunoglobulin domain, a PEVK region (abundant in the amino acids proline (P), glutamate (E), valine (V), and lysine (K)), and an N2A region (141). Different skeletal muscles express different isoforms of titin, with large variations in length observed in the N2A and PEVK regions (139, 143). These different expressions correlate with the mechanical properties of different muscle types (133, 139, 140). Fast skeletal muscles (containing predominately high proportions of fast myosin heavy chains [MHCs]) express short titin N2A isoforms. In contrast, slow skeletal muscles express longer N2A isoforms. Considering this, a functional implication might be that fast muscles are more prone to show an increase in titin-induced force generation during and after stretch contractions compared to slow muscles (Fig. 8, cf. black vs grey trace) (79, 113, 139). Titin in skeletal muscle is known to become stiffer upon muscle activation in the presence of Ca^2+^ (≈ 20%) (59, 144–146). More important, however, seems to be the property to contribute to spring-like force generation during an active stretch from any resting length, likely by attaching to actin during Ca^2+^ activation. This filamentous spring protein titin is known to significantly reduce its persistence length upon activation (147–150). Novel three-filament model approaches have been proposed that explain mechanisms underlying eccentric loading in skeletal muscle based on an adjustable titin spring (26–29). These approaches are backed up by a large number of experimental evidence for titin-actin interactions upon muscle activation (148, 151–154). In particular, a recent study by Dutta et al. (148) demonstrated titin-N2A interaction with actin upon Ca^2+^ activation. This interaction was likely impaired in muscles from muscular dystrophy with myositis (mdm) mice exhibiting an 83 amino acid deletion at the N2A-PEVK intersection, resulting in no increase in titin stiffness and reduced RFE (148).

iii. *Posttranslational modifications of titin*

It is widely known that titin stiffness plays a fundamental role in regulating muscle performance in cardiac and skeletal muscle (133). Alterations in titin stiffness affect the contractile properties of the muscle—particularly during eccentric muscle actions. Several posttranslational modifications of titin mediate the rapid modulation of titin stiffness. The stiffness of titin can be acutely modulated by Ca^2+^ (144) and chaperone binding (155), titin-actin interaction (148, 151–154), and oxidation (156, 157). Another mechanism that contributes to the modulation of titin stiffness is regulated by phosphorylation (158, 159). The modulation depends on the location where protein kinases phosphorylate the elastic titin regions. At both ends of the titin molecule, the phosphorylation status regulates the binding of titin to many Z-disc and M-band proteins (159). The majority of the phosphorylation studies has been done on the cardiac N2B and PEVK elements (160, 161). There is general agreement that phosphorylation of the cardiac N2B region increases the persistence length of the elastic titin spring, which results in reduced overall titin-based stiffness and force. Whereas phosphorylation of the cardiac PEVK domain reduces the effective free spring length yielding increased stretch-dependent stiffness and force. Only a few studies have investigated the phosphorylation of the two titin domains N2A and PEVK in skeletal muscle (162–165). The findings reveal a titin modification detected in eccentrically exercised skeletal muscles of adult rats, resulting in an overall increase in titin-based stiffness (164, 165).

These results suggest that titin’s posttranslational modifications in cardiac and skeletal muscles may act differently upon exercise-induced mechanical stress. The observed changes in titin-based stiffness are thought to play an important role in adjusting the passive and active properties of cardiac and skeletal muscle in health and disease. For detailed reviews on posttranslational modifications of titin see (133, 158–160).

## Future challenges in muscle modelling

A precise knowledge of molecular and cellular mechanisms underlying eccentric contractions is also required for the improvement of muscle models. Muscle models—designed to facilitate realistic predictions of muscle force production during dynamic contractions over the entire working range of the muscle—are used to answer a variety of questions in biology, medicine, biomechanics and physiology (166–168). A precise prediction of muscular forces is needed to gain detailed knowledge of (*i*) the structure and functioning of the muscle, (*ii*) neuromuscular relationships in locomotor systems, (*iii*) the optimization of medical diagnostic and/or treatment methods, but also (*iv*) to address unresolved issues related to mechanical/metabolic movement principles or physiological processes. By modelling titin as a viscoelastic spring segment with an activation-dependent coupling to actin, computational models will be able to mimic active lengthening experiments in biological muscle. A three-filament model of skeletal muscle force production (mediated by titin) is supposed to overcome significant deviations between experimental observations and predictions by the classic sliding-filament (3, 4) and cross-bridge theories (5) (two-filament models). This will improve the accuracy of muscle models (27) as well as multi-body models (169) concerning the control of movements and efficiency of locomotion.

Hence, the development of data-driven numerical methods for the simulation of biological systems (hollow organs [stomach (170, 171), urinary bladder (172, 173)], and skeletal muscles (174)) are of great importance. Due to novel research approaches together with computer simulations, possible binding mechanisms (e.g. actin-titin, titin-tropomyosin) can be tested. The predictive power of complex 3D muscle models is only as good as the physical accuracy comprising each of its components, generally the properties of certain muscle structures, boundary conditions, and/or underlying geometries (169). Such predictions also depend on the correct characterisation of their smallest unit—the (half-) sarcomere. Errors in their description inevitably lead to deviations of the muscular force and thus to issues and misinterpretations of all model-based research. Consequently, the prediction of realistic muscle forces in dynamic contractions allows a better understanding of e.g. overall muscular force production, functional morphology, mechanical principles of locomotion, prosthetics, and robotics or provides a detailed insight into the functionality and motility of hollow organs.

## Conclusion and future perspectives

The long-standing problem in muscle physiology of how muscles operate in eccentric contractions has been intensely studied for the past 70 years—with remarkable progress. Skeletal muscle behaviour during and following active stretches is associated with increased performance at decreased oxygen consumption, reduced metabolic cost (ATP), improved energy efficiency, as well as decreased muscle activity (175–178). Stretch-induced force potentiation exists during voluntary contractions and is relevant for movement generation in daily activities (179). The versatile and unique characteristics driven by eccentric muscle loading represent important determinants of active force production. The progressive force development and linear spring-like muscle behaviour during stretch contractions are not accounted for in existing Hill- or Huxley-type muscle models so far, and might significantly reduce the control effort. Additionally, this distinct manner might offer high-impact shock absorption strategies during eccentric movements such as landing after jumps or downhill running (30). The features discussed above could be demonstrated at the small microscopic scale up to the gross macroscopic scale. Ample evidence supports the idea of a cumulative mechanism that combines non-linear XB and non-XBs contributions to result in a linear force response during muscle-lengthening contractions. Findings suggest that titin is a fundamental regulator in eccentric loading in striated muscle, although its role is still evolving. The mechanical properties of titin continually adapt to cover prevailing conditions of skeletal muscle performance. For all mechanisms noted above, modulation in titin-based stiffness plays an essential role. Titin stiffness alters as a function of titin-isoforms (139), other sarcomeric proteins (such as molecular chaperones) targeting the titin springs (155), the interaction of titin-spring elements with the thin filament actin (26), phosphorylation-mediated regulation (158, 160), and mechanosignalling (120). Current and future findings are likely to improve the understanding of overall muscle behaviour and force generation on different scales touching health, rehabilitation, physical and applied sciences, robotics, and foundations of muscle contraction.

## Data Availability

This is a review paper. The results reported here have been published previously (see references for specifics).
